# Penile gangrene in diabetes mellitus with renal failure: A poor prognostic sign of systemic vascular calciphylaxis

**DOI:** 10.4103/0970-1591.32081

**Published:** 2007

**Authors:** Mayank Mohan Agarwal, Shrawan K. Singh, Arup K. Mandal

**Affiliations:** Department of Urology, Postgraduate institute of Medical Education and Research, Sector 12, Chandigarh - 160 012, India

**Keywords:** Calciphylaxis, gangrene, penis

## Abstract

Penile gangrene associated with chronic renal failure is very uncommon. A 52-year-old man with diabetes mellitus, diffuse atherosclerosis, ischemic cardiomyopathy and end-stage renal disease presented with blackening of distal penis for 10 days. His general condition was poor and gangrene of prepuce and glans was noted. Doppler and magnetic-resonance angiography revealed bilateral internal iliac artery obstruction. He underwent trocar suprapubic cystostomy and was planned for partial penectomy. But he died of severe diabetic complications in the interim period. Penile gangrene is a manifestation of widespread vascular calcifications associated with end-stage renal disease and is a marker of poor prognosis.

## INTRODUCTION

Penile gangrene is very uncommon due to its extensive blood supply. There are case reports of penile gangrene resulting from various causes like priapism, pyoderma gangrenosum, Fournier's gangrene, venous thrombosis, warfarin therapy, systemic vasculitis, constriction due to metallic ring or condom catheter, penile prosthesis, diabetes mellitus and uremia, etc.[1–2]

Gangrene of penis in uremics is a rare occurrence and has been associated with poor prognosis. It is believed to be a manifestation of widespread metastatic vascular calcification attributable to secondary hyperparathyroidism (calciphylaxis)[2] which more commonly manifests as distal extremity gangrene. Treatment is controversial and ranges from observation to parathyroidectomy along with penectomy.

We present a case of a hemodialysis-dependent diabetic patient who presented with subacute penile gangrene and rapidly succumbed to the disease. We also discuss various treatment options in these cases.

## CASE REPORT

**Figure 1:**
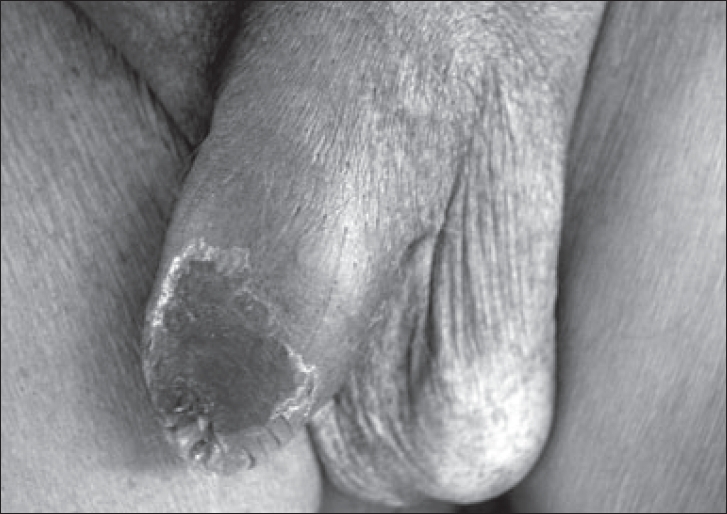
Clinical photograph of the patient's genitalia showing blackening of distal half of penile skin and glans suggestive of gangrene

**Figure 2:**
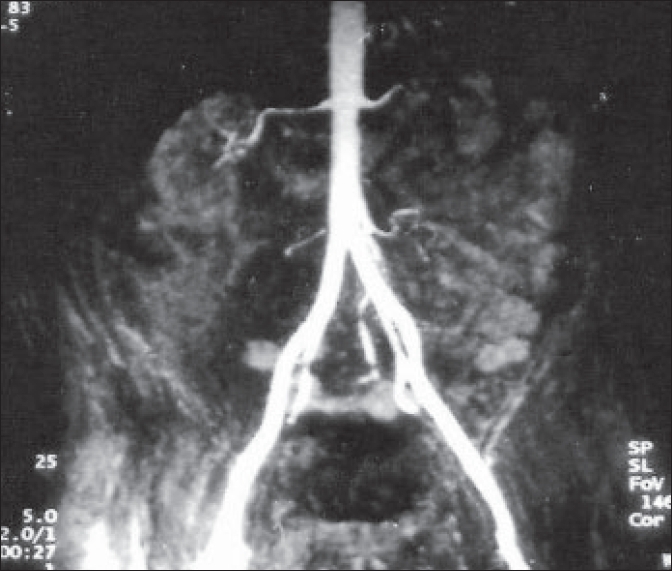
Magnetic resonance angiography showing critical narrowing of bilateral internal iliac arteries

A 52-year-old insulin-dependent diabetic man presented with blackening and pain in distal penis and nonretractability of prepuce of 10-day duration. He was a known case of diabetes mellitus for 10 years and had become insulin-dependent for the last two years. He was also affected by severe diabetic complications, including peripheral neuropathy and nephropathy leading to end-stage renal disease (ESRD) with daily urine output of less than 300 ml and was hemodialysis-dependent for the last one year. He was hypertensive and had developed ischemic cardiomyopathy with low ejection fraction (30%). He was also suffering from atherosclerotic vascular disease and underwent below-knee amputation for gangrene one year back. On examination, he was pale and had generalized edema. His blood pressure was 160/100 mmHg in both arms and bilateral femoral pulses were feeble. Local examination revealed edema of penile skin with blackening of distal half of penile skin and glans suggestive of gangrene [Figure 1]. Investigations revealed hemoglobin of 8 g/dL, total leucocyte count of 12,000/μL, serum creatinine 6.8 mg/dL, blood sugar 180 mg/dL, calcium 8.5 mg/dL, inorganic phosphate 7.5 mg/dL and serum albumin 3.0 mg/dL. Doppler ultrasound revealed poor flow to penile vessels and magnetic resonance angiography [Figure 2] showed critical narrowing of bilateral internal iliac arteries.

He was treated with antibiotics and local hygiene and underwent percutaneous suprapubic cystostomy. He was planned for partial penectomy but his overall condition deteriorated and consequently died of multisystem complications.

## DISCUSSION

An extensive collateral blood supply to the penis protects this organ from ischemic gangrene. This is unlike the fingers, toes, lower limbs and heart which are more prone to ischemic damage due to end-arterial blood supply. Isolated penile gangrene is believed to be a focal manifestation of diffuse arterial calcification seen in chronic renal failure and diabetes mellitus further aggravates the situation. It is extremely rare and only 34 cases have been reported in the world literature. Cholesterol crystal embolism has been reported as another cause of this condition.[3] Rarely, diabetic vasculopathy (including microangiopathy) may also directly lead to penile gangrene in the absence of the above mentioned phenomena, especially in the presence of infection.[4] There was no evidence of infection in the index case.

Calciphylaxis is a disease of smaller arteries, arterioles and capillaries. The larger arteries responsible for palpable pulses are usually spared. It is a manifestation of abnormal calcium and phosphorus metabolism due to secondary hyperparathyroidism that occurs in renal failure patients, especially those on maintenance dialysis.[2] The risk of this metastatic calcification (calciphylaxis), which also occurs in soft tissues, is higher when [Ca^2+^] × [PO_4_^3−^] product exceeds 70 mg^2^/dl^2^; the normal range being 20.6–52.5.[2] The product was 70.7 in the index case. In this process, calcium is deposited in a hypersensitive environment (due to presence of hyperparathyroidism, calcium and phosphate) in response to a challenging agent (e.g., iron overload, Vitamin D, etc.). Calciphylaxis has also been reported in the absence of hyperparathyroidism and other hypercalcemic states, e.g., milk-alkali syndrome, rickets, infantile hypercalcemia, collagen disease, leukemia, lymphoma and multiple myeloma.[2]

The histological appearance is characterized by marked luminal compression owing to calcific infiltration of tunica media and hyperplasia of intima. This is in contrast to the atherosclerotic process in which there is atheromatous replacement of the intima. Nevertheless, atherosclerosis and calciphylaxis can coexist. We could not obtain histopathological specimen in our case due to his sudden demise before planned surgery.

Treatment of penile gangrene associated with calciphylaxis is controversial and varies from subtotal parathyroidectomy with partial or total penectomy to conservative management. In the conservative approach, which is essentially watchful waiting with or without local debridement and suprapubic urinary diversion, penectomy is advised if there is progression of gangrene and sepsis is observed. At the other end parathyroidectomy has been shown to portend some survival advantage and is indicated in patients in whom progressive calcification develops despite medical therapy.[2] It is best performed before extensive necrosis, gangrene and sepsis develop. Lately, intracavernosal prostaglandin injection has been used successfully in a diabetic patient with uremia.[5]

The mortality rate in patients with calciphylaxis due to renal failure remains high despite aggressive management due to severity of associated systemic illness. Therefore, prevention of calciphylaxis appears to be the key factor. As a step to the prevention of calciphylaxis, secondary hyperparathyroidism needs an early aggressive management. Oral supplementation with elemental calcium and vitamin D, oral phosphate binders and dialysis with solutions containing low phosphate and high calcium concentration maintain a low [Ca++] × [PO4−] product. The index case had multiple systemic illnesses and showed progression despite appropriate medical management portending a poor prognosis; he had sudden death probably due to acute cardiac event.
